# Idiopathic calvarial thinning mimicking en coup de sabre deformity

**DOI:** 10.1016/j.jdcr.2025.12.021

**Published:** 2025-12-17

**Authors:** Lucie Joerg, Joseph Schwartz

**Affiliations:** a Albany Medical College, Albany, New York; b J Schwartz MD, PLLC, Troy, New York

**Keywords:** en coup de sabre, idiopathic calvarial thinning, linear scleroderma, morphea, skull bone thinning

## Introduction

Calvarial thinning refers to progressive loss of thickness of the upper, dome-shaped portion of the skull, including the frontal, parietal, and occipital bones. Calvarial thinning may arise from congenital syndromes or as a secondary manifestation of systemic disease, including primary and metastatic tumors, Gorham–Stout disease, hyperparathyroidism, systemic mastocytosis, granulomatosis with polyangiitis, diabetes mellitus, osteomyelitis, cystic angiomatosis, chronic steroid use, and bone aneurysm.^1^ When these etiologies are excluded, the process is termed idiopathic calvarial thinning, a rare, acquired, and indolently progressive reduction in calvarial thickness without antecedent trauma, systemic disorder, congenital syndrome, or surgical intervention.^2^ Radiographically, idiopathic calvarial thinning often presents as localized attenuation, classically with bilateral, symmetric involvement of the parietal bones in older women. Only approximately 150 cases have been reported to date.^3^ The disease pathogenesis, etiology, and optimal treatment guidelines are largely unknown.

Given the rarity of this condition, idiopathic calvarial thinning may be mistaken for morphea, or localized scleroderma, a sclerosing inflammatory skin disease. Morphea is typically categorized into 5 subtypes: linear, circumscribed (plaque), generalized, pan-sclerotic, and mixed forms.^4^ When linear morphea involves the head and neck, it is termed “*en coup de sabre*” (ECDS) or Parry-Romberg syndrome, typically presenting as atrophic and hyperpigmented plaques with a characteristic saber-cut depression or progressive hemifacial atrophy, respectively.^4^ Here, we describe a rare case of idiopathic calvarial thinning mimicking ECDS.

## Case report

**Fig 1:**
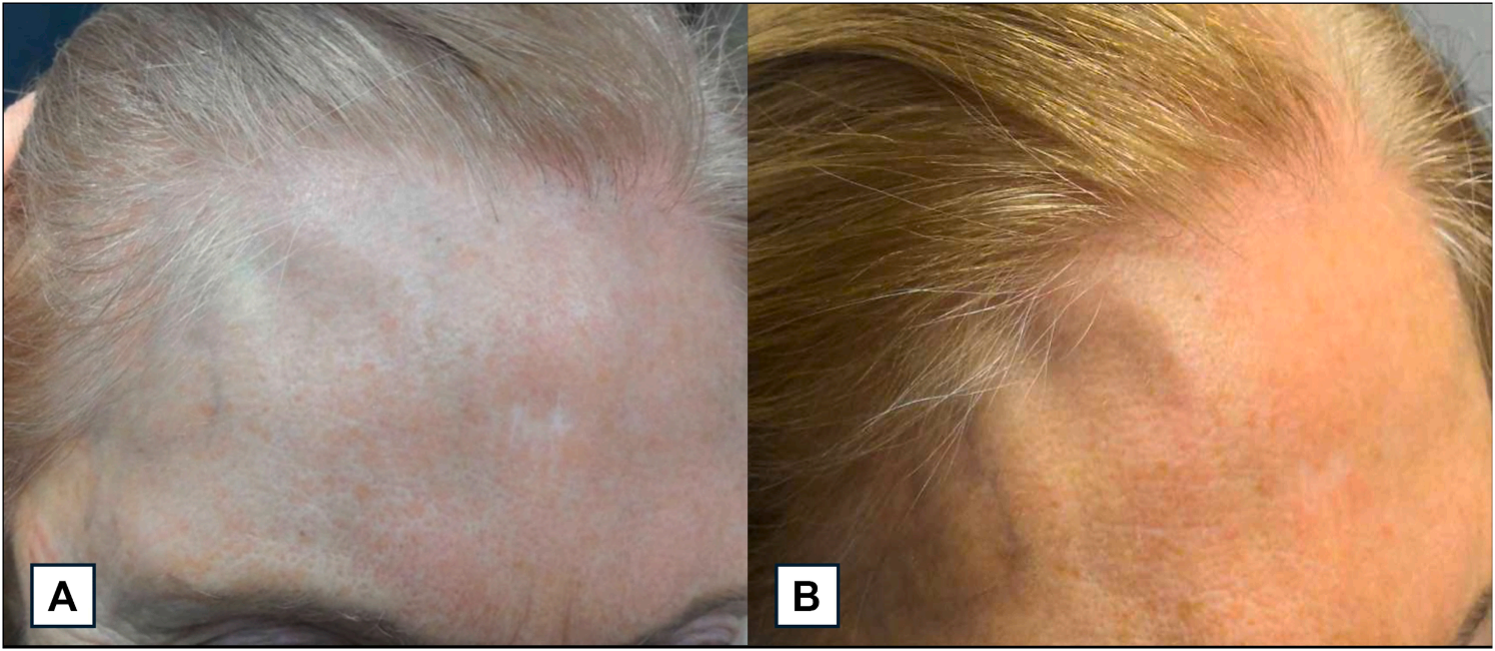
Calvarial thinning. **A,** Initial physical examination of a woman with right frontal bone loss. **B,** Most recent photograph, taken 3 years after Fig 1 *A*, demonstrates visually worsening calvarial thinning.

A 66-year-old female presented to the dermatology clinic for a routine skin screening with a clinical depression on the right forehead that had progressed over 3 years (Fig 1). The patient denied any associated pain, pruritus, or skin tightness. There was no extracutaneous involvement, such as fatigue, arthralgia, myalgia, or Raynaud’s phenomenon. Her past medical history of osteoporosis, subclinical hyperthyroidism, verruca, herpes viral breakout on the buttock and lip, actinic keratosis, and recession of the frontal hairline was noncontributory. The patient had no history of local procedures or prior bisphosphonate use. Her physical examination revealed a single, rounded 2 cm long depression along the right forehead with slight thinning of the associated hairline and concave skull lesions over the bilateral parietal skull (Fig 1, *A*). These foci were anatomically separate and noncontiguous. Cutaneous examination demonstrated a faint, linear hypopigmented scar along the midline forehead, corresponding to a skating injury in 2020 that primarily resulted in a surgical neck fracture of the right humerus; a brain magnetic resonance imaging (MRI) at that time and subsequent evaluations to date revealed no calvarial abnormality in the scar region. The patient did not demonstrate any limb length discrepancy, joint contractures, or decreased range of motion. Her facial muscle strength and neurologic function were preserved.

Following multidisciplinary evaluation with neurosurgery, hematology, rheumatology, and endocrinology, the patient received extensive blood work and imaging, including computed tomography (CT) and MRI. Laboratory results showed negative antinuclear antibodies, anti-SS-A, anti-SS-B, anti-Scl-70 antibody, and anti-Jo antibodies. The complete metabolic panel, including calcium and alkaline phosphatase, as well as the complete blood count with eosinophil count, creatine kinase, C-reactive protein, rheumatoid factor, and urinalysis, was unremarkable.

**Fig 2:**
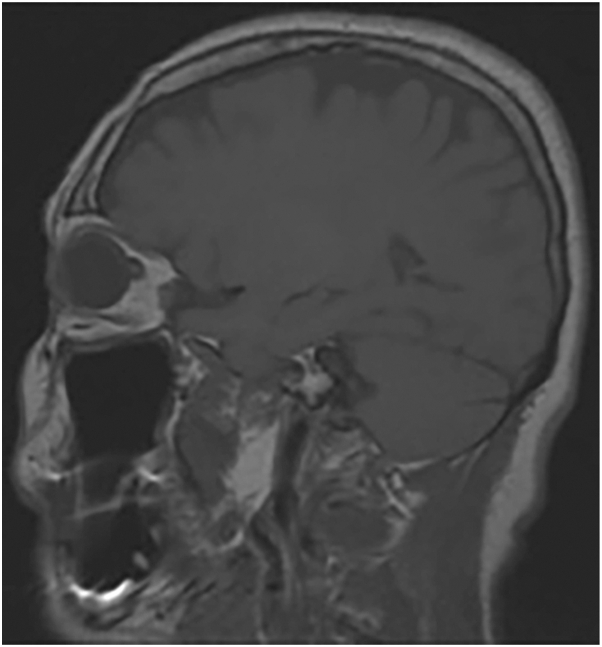
Sagittal view of the brain MRI demonstrating thinning of the right frontal calvarium. *MRI*, Magnetic resonance imaging.

**Fig 3:**
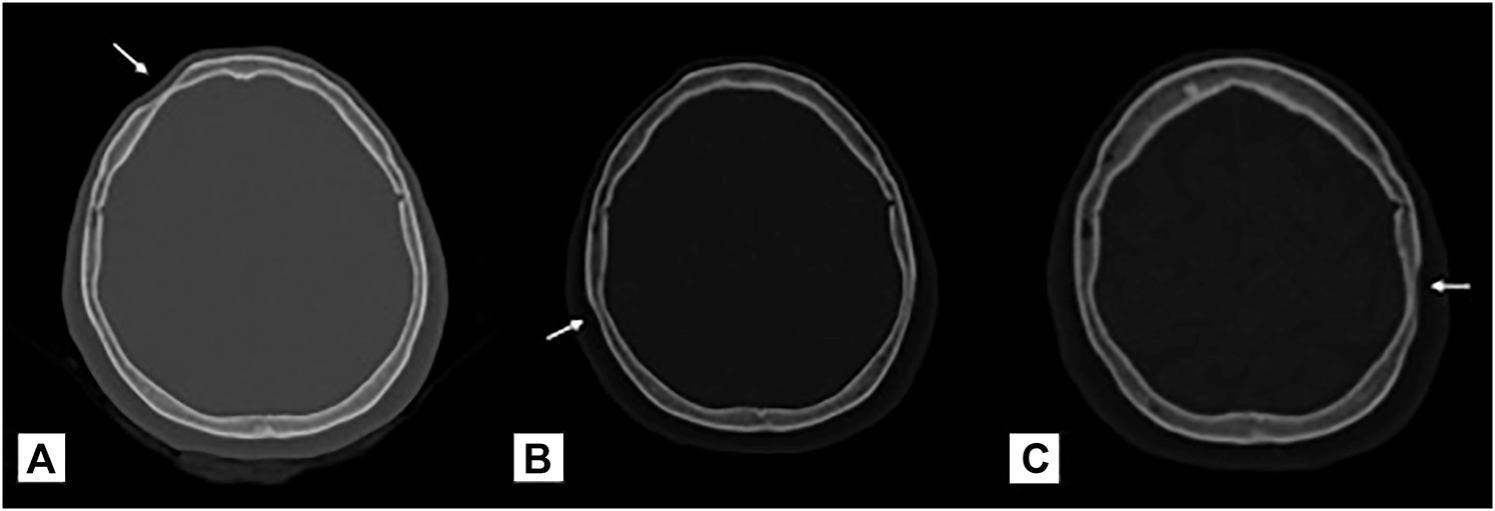
Axial brain CT demonstrating multifocal calvarial thinning. **A,** Discrete, noncontiguous right frontal calvarial thinning with near obliteration of the diploic space and inward contouring (*arrow*). **B,** Subtle shallow depression of the right parietal calvarium with some diploic narrowing (*arrow*). **C,** Focal inward depression of the left parietal calvarium with moderate to severe diploic space narrowing (*arrow*). *CT*, Computed tomography.

**Fig 4:**
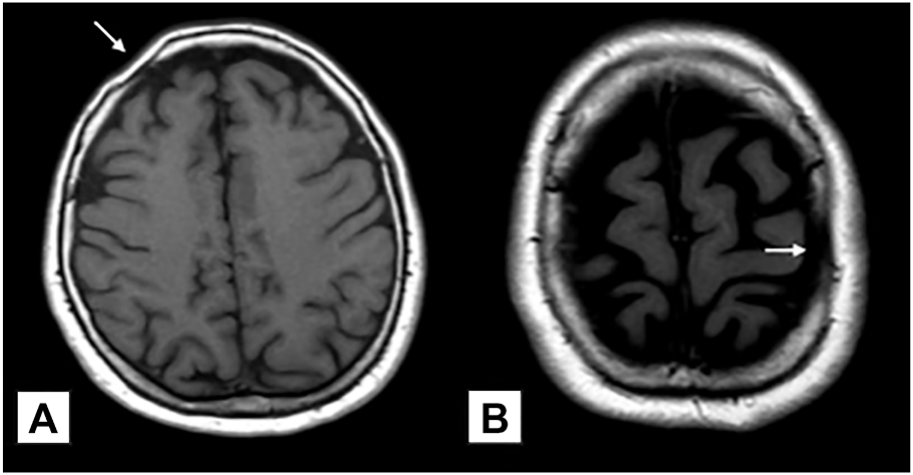
Axial brain MRI revealing severe bone loss. Subcutaneous fat is intact, favoring the diagnosis of idiopathic calvarial thinning. **A,** Right frontal bone thinning of the calvarium (*white arrow*). **B,** Left parietal bone thinning of the calvarium (*white arrow*). *MRI*, Magnetic resonance imaging.

An initial brain MRI in 2022 revealed thinning of the diploic space of the right frontal calvarium with an associated thinning cavity along the inner and outer tables of the calvarium (Fig 2). There was no underlying marrow lesion, intracranial abnormality, or atrophy of the right side of the face to suggest progressive facial hemiatrophy. Over 3 years of surveillance, subsequent MRI and CT demonstrated frontal lesion progression with new foci of calvarial thinning, predominantly involving trabecular bone. Head CT in 2023 redemonstrated right frontal calvarial thinning with near-complete loss of the diploic space (minimum residual thickness, 2 mm) and a concordant inward scalp depression (Fig 3, *A*). Additional shallow, short-segment defects involved the left anterior parietal and right parietal calvarium with outer-table and diploic thinning (Fig 3, *B* and *C*). At the patient’s most recent examination, the right frontal scalp depression had enlarged (Fig 1, *B*). MRI in 2025 revealed marked diploic thinning involving the right frontal and left parietal calvarium (Fig 4, *A* and *B*). No marrow-replacing process, intracranial lesion, or overlying soft-tissue abnormality was identified.

## Discussion

We present a rare case of idiopathic calvarial thinning mimicking ECDS. Although idiopathic calvarial thinning classically involves the bilateral parietal bones, the patient initially exhibited right frontal involvement, progressing to bilateral parietal thinning.^5^ Owing to the presentation’s rarity and atypical features, the diagnostic course was prolonged. Following evaluation by the aforementioned specialties, multidisciplinary consensus supported the final diagnosis. Prior reports of idiopathic calvarial thinning describe gradual progression and even fatal outcomes from calvarial perforation and neurologic consequences, so timely diagnosis and monitoring are warranted. Severe cases require neurosurgical management, including cranioplasty or bone grafting, to restore calvarial integrity and prevent cerebrospinal fluid leakage.^1^

The differential diagnosis included ECDS, a clinical diagnosis established by hallmark cutaneous and soft-tissue features.^6^^,^^7^ Classically, ECDS develops in childhood or early adulthood as linear, frontoparietal sclerotic plaques of the face or scalp, associated with alopecia and soft-tissue atrophy.^6^ Additionally, a meaningful subset of patients demonstrates intracranial abnormalities on CT and MRI, including outer diploe thinning, white matter lesions, cerebral atrophy, and subcortical calcifications, underscoring the potential for extracutaneous involvement.^6^ No disease-specific laboratory tests exist; however, autoantibodies may be present, including antinuclear antibody, anti-single-stranded DNA, anti-double-stranded DNA, anti-histone, anti-topoisomerase IIα, anti-phospholipid, and rheumatoid factor, which can precede disease onset.^8^ Positivity for Scl-70, anticentromere, Ro/La, or U1RNP also warrants close follow-up for potential systemic involvement.^6^

A bone biopsy was not performed, as literature supports an imaging-based approach as the gold standard for biparietal osteodystrophy diagnosis; when clinicoradiologic findings are concordant, invasive sampling is unnecessary.^9^ As no test definitively excludes localized scleroderma, and its variants can cause calvarial thinning without cutaneous changes, morphea localized to the bone cannot be conclusively excluded.^10^ However, the absence of cutaneous sclerosis or subcutaneous atrophy, seventh-decade onset, unremarkable laboratory results, and radiological studies without intracranial or soft-tissue abnormalities favor idiopathic calvarial thinning.

This case highlights how progressive, frontal bone-predominant idiopathic calvarial thinning can mimic ECDS. Careful clinicoradiologic correlation and targeted exclusion of diagnostic look-alikes enable a biopsy-sparing diagnosis and inform ongoing surveillance.

## Conflicts of interest

None disclosed.
